# EGFET-Based Sensors for Bioanalytical Applications: A Review

**DOI:** 10.3390/s18114042

**Published:** 2018-11-20

**Authors:** Salvatore Andrea Pullano, Costantino Davide Critello, Ifana Mahbub, Nishat Tarannum Tasneem, Samira Shamsir, Syed Kamrul Islam, Marta Greco, Antonino S. Fiorillo

**Affiliations:** 1 Department of Health Sciences, University “Magna Græcia” of Catanzaro, 88100 Catanzaro, Italy; critello@unicz.it (C.D.C.); marta.greco@unicz.it (M.G.); nino@unicz.it (A.S.F.); 2 Department of Electrical Engineering, University of North Texas, Denton, TX 76203, USA; Ifana.Mahbub@unt.edu (I.M.); NishatTarannumTasneem@my.unt.edu (N.T.T.); 3 Department of Electrical Engineering and Computer Science, University of Missouri, Columbia, MO 65211, USA; sshamsir@mail.missouri.edu (S.S.); islams@missouri.edu (S.K.I.)

**Keywords:** EGFET, ISFET, electrochemical cell, enzymatic biosensor, DNA–DNA biosensor, immunosensor, antigen–antibody biosensor, ionic sensor, chemosensor

## Abstract

Since the 1970s, a great deal of attention has been paid to the development of semiconductor-based biosensors because of the numerous advantages they offer, including high sensitivity, faster response time, miniaturization, and low-cost manufacturing for quick biospecific analysis with reusable features. Commercial biosensors have become highly desirable in the fields of medicine, food, and environmental monitoring as well as military applications, whereas increasing concerns about food safety and health issues have resulted in the introduction of novel legislative standards for these sensors. Numerous devices have been developed for monitoring biological processes such as nucleic acid hybridization, protein–protein interaction, antigen–antibody bonds, and substrate–enzyme reactions, just to name a few. Since the 1980s, scientific interest moved to the development of semiconductor-based devices, which also include integrated front-end electronics, such as the extended-gate field-effect transistor (EGFET) biosensor, one of the first miniaturized chemical sensors. This work is intended to be a review of the state of the art focused on the development of biosensors and chemosensors based on extended-gate field-effect transistor within the field of bioanalytical applications, which will highlight the most recent research reported in the literature. Moreover, a comparison among the diverse EGFET devices will be presented, giving particular attention to the materials and technologies.

## 1. Introduction

The earliest example of a solid-state device for the sensing of ionic activities can be traced back to 1970 with the ion-sensitive field-effect transistor (ISFET), derived from an insulated-gate field-effect transistor (IGFET) [1]. In this class of devices, the gate consists of only a thin SiO_2_ layer in contact with an electrolyte solution contained in an electrochemical cell, and the potential at the interface electrolyte/solution influences the drain current [1,2]. The device and the electrical connections are separated from the solution, avoiding possible damage caused by the penetration of liquids, which guarantees insulation from the external environment as well as biocompatibility. The information retrieved from the sample, which in the first approximation is the analyte concentration, depends on the interfacial potential with respect to an external reference electrode, which is part of the sensor itself. It provides a known, stable potential that does not depend on the intensity of the current (i.e., zero-current condition). The development of extended-gate field-effect transistor (EGFET) sprang from ISFET technology, and was first proposed by Van der Spiegel in 1983 [3].

Unlike ISFET, EGFET preserves the gate region as a standard metal-oxide-semiconductor field-effect transistor (MOSFET) and the sensing membranes are physically separated [4,5,6]. Thus, the activity of target analyte results in an additional chemical contribution to the threshold voltage (*V_th_*) [7]. The main applications of EGFETs are associated with the detection of ionic species, pH, and specific molecules (through the functionalization of sensitive surfaces) such as urea and glucose [7,8,9,10,11,12]. Apart from its prevalent use in the fields of biosensing and chemical sensing, physical sensors for high-frequency ultrasound detection (e.g., hydrophones) have been realized through EGFET technology, often referred to as piezoelectric-oxide-semiconductor field-effect transistors (POSFETs) or piezoelectric gate on FETs (PiGoFETs) [13,14,15].

The foremost problem with these solid-state devices is threshold voltage drift, which depends on the chemistry of the environment under test [16,17]. This phenomenon manifests itself through a slow, continuous change in the threshold voltage of the device, usually in one direction. Different technical solutions have been proposed for its improvement, such as encapsulation techniques, even though there is not yet any general technology for the poor isolation between the device and the chemical environment [18]. The most diffused involve multilayer materials, such as the backside gate type with Si–SiO_2_–Si (SIS) [19] or silicon-on-sapphire (SOS) structure [20,21]. Other techniques include the use of polymeric membranes [22], photoresist [23], glass-bonded [24], and epoxy resin encapsulation [25]. Even though a great deal of effort has been made toward solving this limiting factor, the effects of drift remain a sticking point [16,26]. Recently, the introduction of microfluidics together with the integration of EGFET biosensors has made the packaging easier, with a reduction in the volume of samples and reagents [27]. The following sections will look more closely at the principal components and relative functioning principles of EGFET, along with their foremost applications.

## 2. Fundamental Principles of EGFET

### 2.1. FET Device

**Figure 1:**
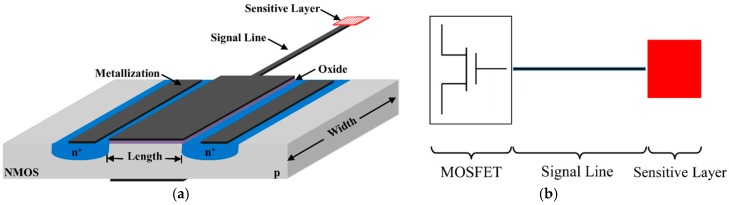
(**a**) Section of a device based on extended-gate field-effect transistor (EGFET) technology (not to scale); and (**b**) schematic of a separative-extended gate-field-effect transistor (SEGFET).

The principal component of an EGFET device is a MOSFET, which confers long-term stability on environmental variations (such as light and temperature), facilitates insulation and encapsulation, and makes it possible to vary the geometry of the sensing membrane more easily than an ISFET [28,29,30,31]. The working principle is that of a conventional MOSFET, except for the sensing layer, which is immersed in a buffer solution positioned at a certain distance from the device [32]. As can be seen in Figure 1a, the device consists of a FET with high input impedance, a signal line (better if shielded), and the sensitive area connected to the gate [3]. EGFET allows the use of both on-chip integrated preamplifiers and, in the most recent configurations reported in the literature, discrete commercial-type devices connected to the gate extension. The latter is more precisely referred to as separative-extended gate-field-effect transistor (SEGFET), as shown in Figure 1b. Sometimes, it is also named ExGFET in order to avoid ambiguity with electrolyte-gated field-effect transistor.

The impedance of the sensitive layer of the EGFET is usually different from the ISFET, in which it coincides with a high-impedance-gate dielectric. The sensitive layer (e.g., redox-responsive material) of an EGFET is instead characterized by low impedance, with consequently higher conductivity and sensitivity [33]. According to the MOSFET literature, the equation that binds the channel current *I_DS_* to the characteristics of the EFGET, usually in the linear region, is as follows:

(1) IDS=μCoxWL[(VRef−Vth∗)VDS−12VDS2] 

where *W* and *L* are the width and length of the channel, *μ* is the mobility, *C_ox_* is the gate oxide capacitance per unit area, and *V_Ref_* and *V_DS_* are the applied reference electrode and the drain-to-source voltages, respectively. In Equation (1), the only parameter linked to the analyte (i.e., the activity of the analyte) is the overall threshold voltage V^*^_th_ of the device [30,32].

The aspect ratio *W*/*L*, and thus the transconductance *g_m_*, influences the performance of the device, particularly the input-referred noise. High *g_m_* values, and consequently devices with a large surface area, reduce the flicker (*1/f*) noise, which is implicated in the surface conduction phenomena of the MOS caused by carrier recombination and interface traps. In fact, the oxide–semiconductor interface is characterized by random trapping and detrapping of the carriers that flow through the channel, generating flicker in the drain current [34]. Since one of the aims of the EGFET is to further reduce the response time, by imposing the minimum possible channel length, the gate width can be modified, increasing the aspect ratio. An empirical model for evaluating the power spectral density (PSD) of the flicker noise is expressed by the following:

(2) $$\overset{\cdot }{S_{I_{DS}}=\frac{Mg_{m}^{2}}{C_{ox}^{2}WL}\frac{1}{f^{\gamma }}}\;$$

where *f* is the working frequency, *M* is an empirical parameter, and *γ* is a process parameter [34]. The PSD increases with the increase of the drain current depending on the region of operation, while large surface positively affects *S_IDS_*. Humidity at high temperature has a different impact on the flicker noise depending on the channel type. Humidity affects flicker noise, mostly for *p*-channel devices, while in an *n*-channel, the contribution is negligible [35]. Therefore, the increase of *W* impacts the performance, especially for low-frequency applications [14]. In addition to the flicker noise, both the device and the transduction interface are intrinsically affected by thermal noise, depending on the leakage current at the oxide interface [14,15]. Thermal noise spectral density has been investigated in MOSFETs by Van der Spiegel in strong, moderate, and weak inversion regions in high-*W*/*L* devices. The proposed model (valid only for “long channel” devices) highlighted that *n*-channel MOSFET is characterized by higher noise due to a larger body effect apart from strong inversion, where the effect becomes less significant with respect to the *p*-channel device [36]. Latch-up and noise reduction can be obtained by proper bulk/well biasing and design layout. Apart from the noise ascribable to the FET, and eventually to the signal line if not properly shielded, the EGFET sensor does not significantly suffer from other external interference due to coupling from high to low impedance, since it is physically located near where the reaction takes place [37].

The possibility of designing ultra-short-channel EGFETs makes it necessary to revise and modify many of the corresponding long-channel counterparts in terms of channel doping, oxide thickness, and potential distribution. Different empirical models have been proposed for adjusting the device parameters preserving the overall MOSFET behavior [38]. The length at which the long-channel behavior is maintained is as follows:

(3) $$L\geq C\sqrt[{3}]{x_{j}t_{ox}{\left(W_{s}+W_{d}\right)}^{2}}\;$$

where *C* is a constant evaluated by fitting Equation (3), *x_j_* is the junction depth, and *W_s_* and *W_d_* are the source and drain depletion depths (that can be subsequently designed), respectively [38]. The integration of a source follower as an input stage together with a bootstrap reduces the input capacitance, which increases the overall sensitivity of the sensor [3]. Concerning the signal line, in the original design, it could be up to several millimeters long, thus the use of an insulating layer allows both electric and chemical shielding, reducing even crosstalk in the case of sensors located near each other [3]. This is even more evident in the most recent EGFET devices, in which the signal line is even longer. Moreover, bootstrapping the shield means that there will be less capacitive coupling between the signal line and the insulating shield, also impacting the bandwidth of the sensor [39]. The threshold voltage V^*^_th_ also depends on the typical parameters of the chemical environment in agreement with the following:

(4) $$V_{th}{}^{*}=V_{th}+E_{Ref}+\chi_{sol}-\frac{W_{M}}{q}-\phi \;$$

where *E_Ref_* is the reference electrode potential, *χ_sol_* is the superficial dipole potential of the electrolyte, *W_M_* is the work function of the reference electrode, *ϕ* is the potential of the surface at the electrolyte/sensing membrane interface, and *q* is the charge [2]. In other devices, such as those exploiting the electrolyte–insulator–semiconductor (EIS) capacitive effect, an external electronic circuit is required to amplify the signal. Moreover, the equivalent capacitive model of the EIS is complex, notwithstanding its simple structure [40].

**Figure 2:**
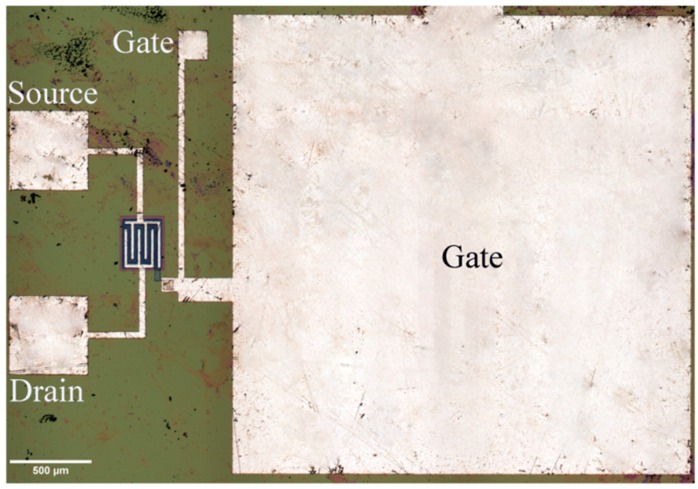
One of the first examples of an EGFET-based sensor realized in the 1980s. It is characterized by an aspect ratio of 1900/5 μm/μm, originally fabricated for an integrated ultrasonic transducer, resulting from a collaboration with the Center for Sensor Technologies, University of Pennsylvania (courtesy of Prof. J. Van der Spiegel) [14].

EGFETs were designed as devices that had to improve the limits, in terms of the output variability of ISFETs, and their performance has been investigated under various conditions such as temperature [41], light exposure [31], and different chemical environments [28]. As compared to ISFETs, they have shown greater chemical and thermal stability as well as better output stability under different incident light conditions, and increased current sensitivity [41,42]. EGFET devices have been widely used in bioanalytical applications for detection of pH, enzymes, and proteins [28,43]. As far as their design is concerned, different topologies have been reported. Figure 2 shows an example of an integrated circuit, in which the noteworthy dimensions of the gate electrode (sensitive area) are evident as compared to the source and drain electrodes.

Signal-to-noise-ratio (SNR) can be used as a performance metric that combines most of the parameters involved during EGFET fabrication, such as *g_m_*, device dimensions, and oxide traps. Rajan et al. pointed out that scaling did not always lead to devices with higher performance. A linear dependence was observed between SNR and √*WL*, which encouraged the design of higher-surface-area EGFETs for improved sensitivity and limits of detection [44]. The design of an optimal gate area rather than gate width is preferred because flicker noise contribution depends on *WL* and is independent of *W*/*L* ratio and the DC bias conditions [45]. As a general rule, for a standard MOSFET, the gate length should be 5–10 times larger than the characteristic length of source-drain lateral potential decays [46]. Van der Spiegel et al. proposed an extended-gate FET with an aspect ratio of 1900/10 μm/μm with the aim of improving gm and reducing the noise [14]. SNR is bias-dependent and thus can be optimized in order to further reduce power consumption [44].

### 2.2. Sensitive Layer

In bioanalytical applications, the potential of specific chemical species involved in the electrochemical reaction is transduced to an electrical signal proportional to the target information (e.g., concentration) [47,48,49,50,51]. Depending on the electrochemical cell configuration chosen, the electrical characterization can be performed through potentiometric, amperometric, or conductometric schemes [52]. The suitability of a particular configuration depends on the specific analyte, the sensitivity and selectivity required, and the design of the overall system. The monitoring of an EGFET-based electrochemical cell requires one or more additional electrodes (i.e., reference and counter electrodes) besides the electrode where the reaction takes place (i.e., working electrode) [52,53]. The two-electrode configuration, the most commonly employed, can set detection limits due to limiting factors (e.g., overvoltage with respect to the zero current half-cell potential) [51]. One alternative solution is to use a three-electrode configuration by adding a counter electrode (e.g., amperometric scheme).

**Figure 3:**
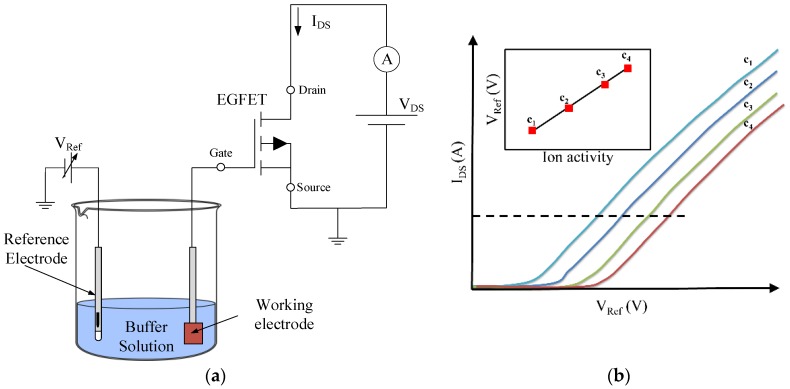
(**a**) Typical setup for EGFET-based potentiometric sensor system. (**b**) Transfer characteristics (*I_DS_–V_Ref_*) of an analyte-sensitive EGFET at different concentrations. The inset shows the dependence of concentration with respect to the reference voltage.

Potentiometric configuration is primarily involved in evaluating the potential across an interface, often a membrane [54,55]. In direct potentiometry, the electromotive force is ideally a function of the activity of a single ion so that it can be selectively evaluated, even in the presence of other species [41]. Most of the EGFETs proposed in the literature exploit the potentiometric configuration, as shown in Figure 3a. The system consists of a working electrode that has a sensitive layer or surface on which the electrochemical reaction takes place. The charge density on the surface of the sensing film will change the surface potential of the sensing film itself, modifying the characteristic curve of the transistor as well. More recently, ion-sensitive devices exploiting floating gates without the use of a reference electrode have been investigated by applying a bias voltage to a control gate rather than a reference electrode [56,57]. Typical characteristic curves for an ion-sensitive EGFET are shown in Figure 3b, in which the current–voltage characteristics of the transistor change depending on the analyte, and the input signal is assessed by the transfer characteristics (*I_D_–V_Ref_*) using a semiconductor parametric device analyzer [41].

Shinwari et al. proposed an in-depth analysis of microfabricated electrodes for biosensors, including those based on FET technology [58]. The most frequently employed electrodes (especially those used as reference) are fabricated in Ag/AgCl because of their smaller size, simpler fabrication, and integration into the device. In addition, these electrodes are not susceptible to the corrosion phenomena of the solution. Other electrodes are hydrogen, saturated calomel electrode (SCE) based on elemental mercury and mercury (I) chloride, copper/copper sulfate, and palladium/hydrogen. However, the reference electrode does not have stable potential depending on the circulating current, but the use of high-input MOS interface minimizes any leakage current that can change its potential [58].

Working electrode materials play an important role in the specific biosensors to be fabricated. Materials can be categorized as metal-based, carbon-based, and polymer-based electrodes. In many cases reported in the literature, the working electrodes are functionalized with nanomaterials and mediators to enhance sensitivity and selectivity [2]. Working electrodes based on metal oxides (insoluble and stable in solution), as discussed in more detail later, have been classically investigated for pH sensors and represent a widespread application involving EGFET devices [4,8,9]. The general approach that is largely accepted is based on the theory of the electric double layer and the electric charge gathered at the oxide surface. It is valid for metal oxides whose charging mechanisms follow the association–dissociation of an amphoteric group [2]. Apart from pH detection, the investigation of sensitive layers functionalized with biological molecules (e.g., enzymes, antibodies, nucleic acids) allows for specific binding or catalytic reactions, resulting in electron transfer related to the specific analyte or a group of analytes (usually oxidases). The functionalized bio-selective surface overcomes the slow kinetics of the target molecules, even though enzyme-free electrodes have also been investigated more recently with good results. There are a variety of available enzymes and each one is appropriate to catalyze an electrodic reaction in the presence of a specific analyte. Table 1 shows a few representative substrates investigated for the fabrication of sensitive surfaces, the related enzymes, the target molecules, and the immobilization techniques adopted. The ability to catalyze a large number of reactions not being consumed, allowing a “continuous” use of the device, is one of the main reasons for the common use of enzymatic biosensors. However, the lifetime of the sensor is limited by the stability of the enzyme.

Recent studies have also reported a further classification of enzymatic sensors into three successive generations of devices: the first, which is limited by nonspecific electroactive particles; the second, in which mediators are employed as electron carriers; and the third, in which there is a direct electron transfer between the electrode and the enzyme (absence of mediators) [80]. In addition to biosensor devices that exploit catalytic enzyme activity, molecular interactions such as antigen–antibody reactions (immunosensors) and nucleic acid interactions (genosensors, also referred to as bioaffinity devices), represent a field of increasing interest for EGFET-based devices [7]. The following sections review the most widespread literature on EGFET, classified for each specific application.

## 3. Applications of EGFET-Based Sensors

### 3.1. pH Sensors

pH is an extremely important biological parameter for human health, providing information for diagnosing many diseases as well as enhancing therapeutic treatment. It can also be used as a tool for monitoring biological and biochemical processes. One of the foremost recent examples concerns tumor cells, for which an elevated pH is an indicator of the onset of cell proliferation [81,82]. Monitoring pH levels of living cells is also important in endocytosis and phagocytosis [83,84]. Among the analytical models developed so far, the most widespread is the *site*-*binding* model*,* which was first introduced by Yates in 1973 (*site*-*dissociation* model) and then further developed by the same group. The model describes the electric double layer at the oxide–electrolyte interface, supposing an amphoteric oxide layer [85]. The pH of the electrolyte solution influences the hydroxyl groups at the surface (i.e., sensing membrane potential) as follows:

(5) $$-2.303\Delta pH=\frac{q\phi }{kT}+\sinh^{-1}\left(\frac{q\phi }{kT\beta }\right)\;$$

where $\Delta pH=pH_{PZC}-pH$, *pH_PZC_* corresponds to the pH value for which the surface charge is null, *k* is the Boltzmann constant, *T* is the temperature, and *β* is the buffer capacity, a parameter that characterizes the ability of the sensitive surface to buffer pH changes (*β* is an inherent property of the sensing material) [86]. According to the site-binding model, the number of binding sites on the sensing membrane can change the potential of the electrolytic interface, as well as the potential of the sensing membrane, as expressed by [87]:

(6) $$\phi =2.303\frac{kT}{q}\frac{\beta }{\beta +1}\left(pH_{PZC}-pH\right)\;$$

The difference in potential between the reference electrode and the sensing membrane is *V_out_* = *V_Ref_* − *ϕ* [88]. The applications of pH sensors are numerous. For example, they are used in the monitoring of drinking water quality, in soil analysis, and in the inspection of processes in the food industry [89,90]. Different oxide substrates have been used as sensing membranes, such as tantalum oxide (Ta_2_O_5_), indium–tin oxide (ITO), tin oxide (SnO_2_), platinum dioxide (PtO_2_), vanadium anhydride (V_2_O_5_), xerogel, niobium oxide (Nb_2_O_5_), zinc oxide (ZnO), titanium dioxide (TiO_2_), and ruthenium oxide (RuO_2_), to name a few [41,42,91,92,93,94,95,96,97,98,99,100,101,102,103]. To further reduce manufacturing costs, particularly of the sensing membrane, recycled materials from other sectors have also been investigated. Industrial-grade touch panel film (TPF) is an example. TPF is a multilayered material composed of ITO/SiO_2_/Nb_2_O_5_ that has several advantages when used as a pH sensor, such as good sensitivity and fast response speed [104]. As already mentioned, the list of materials used as sensing membranes is very long, and the choice depends mainly on the range of interest and the required sensitivity [95,104,105,106,107,108,109]. Most of the efforts reported in the literature have been aimed at improving the performance of EGFET-based pH sensors with the use of different materials, including composites. The buffer capacity *β* has been intensively investigated to improve sensor sensitivity. According to the literature, it can be expressed as:

(7) $$\beta =\frac{2q^{2}N_{s}\sqrt{K_{a}/K_{b}}}{kTC_{d}}\;$$

where *N_s_* is the surface site numbers, *K_a_* and *K_b_* are equilibrium constants, and *C_d_* is the differential capacitance of the electrolyte, which accounts for the storage of electric charge after experiencing electrostatic potential [87]. The buffer capacity and the differential capacitance mostly affect the pH sensitivity of the sensor, leading to a maximum sensitivity for *β* >> 1, which is limited to 59 mV/pH at 297 K (the so-called Nernst limit) [110,111,112]. Nevertheless, studies have reported materials showing super-Nernstian sensitivity [113]. Table 2 shows a few representative pH sensors based on EGFET with particular emphasis on the materials technology, the FET device, and the overall biosensor characteristics.

As shown, there are many usable sensitive layers that can facilitate the development of even more tailored sensor solutions for specific applications. Furthermore, EGFET-based sensors can be miniaturized and suitably integrated into different microsystems [118].

### 3.2. Urea Sensors

Urea is generally known as the best indicator for evaluating the level of uremic toxins in the bloodstream. Different types of biosensors have been proposed for urea detection, particularly based on ISFET and enzymatic field-effect transistor (EnFET) [8,119,120]. Urea sensors are widely used in medical diagnostics for pathologies such as kidney failure, leukemia, diabetes, and hyperthyroidism [121]. Urease is the specific immobilized enzyme used for the detection of urea, following the reaction:

(8) $${\mathrm{CO}(\mathrm{NH}}_{2}{)}_{2}{+3H}_{2}O\overset{\mathrm{urease}}{\to }{2\mathrm{NH}}_{4}^{+}{+\mathrm{HCO}}_{3}^{-}{+\mathrm{OH}}^{-}\;$$

Urease catalyzes the hydrolysis of urea, which can be determined through a change in pH or the concentration of ammonium ions. Urease is immobilized on the sensing membrane by different methods, such as physical adsorption, entrapment, covalent bonding, and cross-linking [122,123,124,125,126]. Lately, in order to simplify the production process and improve the efficiency of enzyme/analyte bonds, urea sensors based on EGFET have been developed [95,127]. Covalent bonding is a very effective method of immobilization due to the strong interaction between the enzymes and the biological material, giving the biosensors longer time stability [119]. Alternative low-cost (inorganic) methods have also been developed, including plasma treatment (e.g., plasma-enhanced chemical vapor deposition) of ITO/polyethylene terephthalate (PET) substrate, which creates amino bonds on the surface and immobilizes urease [86,94]. Chen et al. developed an urea sensor based on EGFET technology using a structure made of tin dioxide (SnO_2_), ITO, and glass as sensing electrodes, characterized by a response time of 1–2 min with high immunity to variations of light and temperature [95]. Besides tin oxide, fluorine doped tin oxide (FTO) has also been used as a sensitive material on EGFET sensors, obtaining a pH sensitivity of 54.10 mV/pH (pH range from 2 to 12) and urea sensitivity of 8.92 µA/pC_urea_ (see also Table 3) [117].

A comparison among EGFET-, ISFET-, and EnFET-based urea sensors shows very similar performance in terms of sensitivity and dynamic range (see Table 3). The majority of EnFETs are based on pH-sensitive FETs, while in some cases they use ISFETs [128]. Pijanowska et al. proposed an ISFET-based urea biosensor for human blood serum with a response time of 80 s and a lifetime of 35 days [8]. Yu et al. proposed a Ta_2_O_5_-based EnFET that had a response time of about 120 s [119]. Concerning EGFET urea sensors, the response time of representative devices is limited to around 2–3 min with a lifetime of >6 days [94,95]. Being of clinical interest (especially with regard to blood), EGFET-based urea sensors represent a promising technology (especially for the development of disposable devices) that indirectly detects urea concentration through the evaluation of pH variation as a result of an enzymatically catalyzed reaction.

### 3.3. Glucose Sensors

The first biosensor for the measurement of glucose was described by Clark and Lyons in 1962 [129]. The principle of detection is based on the generation of gluconate and hydrogen peroxide (H_2_O_2_) catalyzed by glucose oxidase enzyme (GOD) in a glucose solution. By means of a dissociation reaction of H_2_O_2_, the potential of the sensing membrane changes. The chemical reaction, catalyzed by the GOD enzyme, is as follows:

(9) $${\mathrm{Glucose}+O}_{2}\overset{\mathrm{GOD}}{\to }\mathrm{Gluconic}\_\mathrm{Acid}+H_{2}O_{2}$$

$$H_{2}O_{2}\to O_{2}+{2H}^{+}+{2e}^{-}\;$$

Zinc oxide (ZnO) is a common material used for the fabrication of sensing membranes. Nanostructured ZnO layers (e.g., micro/nanowires, nanotubes, and nanonails) fabricated at low temperature (<100 °C) or high temperature (e.g., 650 and 960 °C) have been widely investigated in glucose detection, showing a linearity greater than 99% and good sensitivity (from 21.7 to 89.74 μA·mM^−1^·cm^−2^) depending on the specific nanostructure [130,131,132,133]. In fact, besides temperature, surface morphology influences the sensing characteristics of the glucose sensors (e.g., sensitivity). One-dimensional ZnO nanostructure layers (e.g., nanotubes, nanorods, and nanonails) have been widely investigated because of their high surface-to-volume ratio, which improves the sensing properties of the sensors [130,131,132,133]. Vertically aligned ZnO nanowires are inherently characterized by a high surface-to-volume ratio. The length and width of ZnO nanowires, which can be controlled during the process, lead to a tunable surface area and thus the device’s sensitivity [134]. In order to increase the conductivity of this membrane, the zinc oxide can be “loaded” with metallic elements. When aluminum is chosen, it is referred to as AZO (aluminum-doped ZnO). Wang et al. proposed an EGFET based on AZO nanostructure fabricated with a low-temperature process that showed promising sensing characteristics (e.g., sensitivity of 60.5 μA·mM^−1^·cm^−2^ and linearity > 99%) for a disposable biosensor [101]. The morphology of AZO nanostructures is related to aluminum content. Well-matched ratios of aluminum and ZnO showed higher crystallinity and better conductivity, resulting in superior sensing characteristics [107]. Due to their clinical interest and an electrically favorable chemical reaction, glucose sensors have been widely investigated on different biological fluids (e.g., blood, saliva, tears, sweat). Table 4 presents the main characteristics of EGFET-based glucose sensors with particular emphasis on materials and performance.

### 3.4. Calcium Ion Sensors

Calcium ions regulate a series of biological processes, such as cell proliferation, gene expression, and apoptosis, through bonding interactions with specific proteins, each one having a different affinity [141]. The proteins that bind with calcium are found both inside and outside of the living cells, although it acts as a “trigger” for the abovementioned processes [142]. Sensing nanostructures based on semiconductors offer advantages in terms of biocompatibility, time response, and miniaturization (see Table 5). Various materials have been used to realize sensing membranes for these sensors. For instance, zinc oxide was used for the realization of nanorod sensors, developed to measure the concentration of intra- and extracellular calcium ions [99,143,144]. These sensors, integrated on FETs with a separate extended gate, have been shown to be linear and sturdy [145].

### 3.5. DNA Sensors

**Figure 4:**
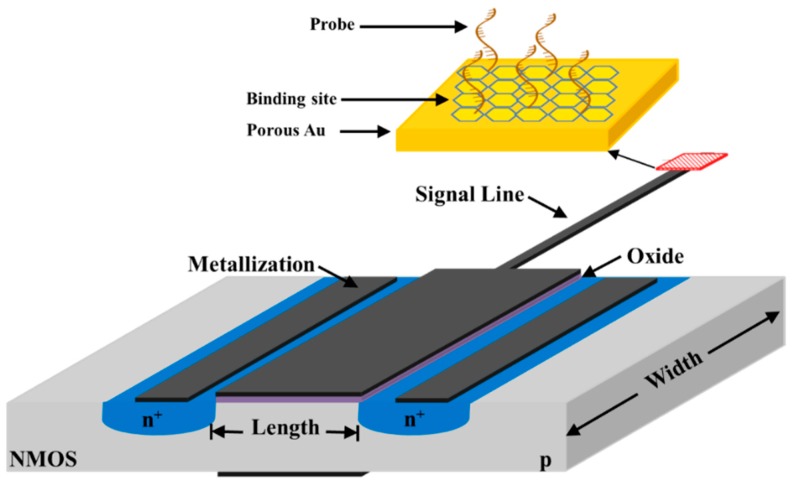
Schematic of an EGFET for DNA sensing using a nanoporous gold layer with binding site (e.g., thiole) for enhancing probe immobilization (not to scale).

Nucleic acid biosensors find wide application in the identification of pathogens, pharmaceutical screening, and the diagnosis of genetic diseases, as well as virus and tumor cell detection. The working principle of these sensors is based on the specific recognition of a single short chain of nucleic acids that hybridizes with a complementary DNA/RNA strand. FET technology for acid nucleic detection is mostly characterized by sensing membranes capable of amplifying the events of nucleic acid hybridization. For instance, an EGFET device with an Au nanoporous membrane has been recently proposed for the evaluation of *Staphylococcus aureus* 16S rRNA (see Figure 4). The presence of this membrane gives the device high linearity, a broad dynamic range (from 10^1^ pM to 10^6^ pM), and a limit of detection of ~1 pM [150]. Sensing membranes based on nanowires and nanofilms have been investigated recently as a part of an extended-gate device. Gallium nitride (GaN) was used because of its biocompatibility and nontoxicity. Both biosensors exhibited high selectivity and rapid detection, although those fabricated with nanowires were more sensitive, showing a wide dynamic range (from 10^−19^ to 10^−6^ M) [151].

### 3.6. Immunosensors

Immunosensors belong to a class of biosensors based on antigen–antibody interactions in biological fluids, a well-recognized and consolidated technology in the field of clinical diagnostics. These biosensors are also used in applications in environmental pollution and food safety when the analytes is a microorganism such as a bacterium, virus, or herbicide. Recently, an immunosensor has been developed and characterized using SEGFET technology for the detection of dengue virus nonstructural protein 1 (NS1) [152]. This sensor consists of the previously discussed separate extended-gate terminal (gold electrode), modified with anti-NS1 antibodies. This device has shown a linear current response for concentrations of less than 1 µg/mL with a detection limit of 0.25 µg/mL. The fabricated device and a similar one, which are reported in the most recent literature, suggest the possibility of using this biosensor as an alternative to the conventional techniques that require adequate instruments and trained personnel.

## 4. Discussion and Conclusions

This review intends to cover most of the recent developments in the field of EGFET-based bio/chemosensors while giving insights into the technological context within which they have been developed. Even though not exhaustive, the work is mainly focused on the solid-state devices used as interfaces of the electronic sensors, the technologies actually investigated for the sensitive layers, and the main related applications reported in the literature. Since the introduction of the first biosensors, such as ISFET and EGFET, the scientific community has been developing even more trustworthy sensing devices that are increasingly responsive to biological and chemical species. As highlighted in the previous sections, development of these devices is currently expanding thanks to the implementation of even more sophisticated nanotechnologies derived from electronics, physics, and materials science. This class of sensors, particularly the potentiometric devices using FET technology, are more easily miniaturized as compared to other types of biosensors (optical, thermal, etc.), justifying the strong scientific interest in the field of biomedical research. In general, the use of FETs allows low-cost transduction of biological information by means of a specific interaction between the sensitive element and the analyte, avoiding cumbersome and complex instruments and reducing chemical reagents and specific markers (label-free). Van der Spiegel highlighted the important points concerning the development of a new class of monolithic sensors utilizing novel FET devices, including the fabrication of a sensitive layer with high specificity for the desired chemical species, placed separately from the transistor to avoid possible damage caused by the surrounding chemical environment. This is what the rapid deterioration of the semiconductor material springs from, along with the resulting problems. In current scientific literature, the terms EGFET and SEGFET involve two different modes of approaching the development of electrochemical biosensors for the detection of analytes. As previously highlighted, since their development, one of the most interesting features of EGFET bio/chemosensors was the possibility of monitoring the binding of species of interest by a direct change in the electrical property of the device. Consequently, an important aspect in the development of high-performance EGFET sensors concerns the selectivity of the device, which allows the detection of analytes while avoiding interference. The selective detection of analytes avoiding contributions from undesired chemical species is a less mentioned but still hot research topic, which is mainly limited by the properties of the interface between the chemical environment and the sensitive layer. The most recent literature reports comprehensive reviews (to refer to for in-depth insights) focused on the engineering of sensitive layers for the production of new biomolecules with tailor-designed properties as a strategy for improving the analytical performance of EGFET [153,154].

Over the years, most of the efforts have been focused on the possibility of applying materials (often insulating materials) in the field of ion sensing, which established well-defined design criteria, from the more classic metal/metal oxide electrodes to the more recent nanostructured materials, not neglecting the use of different materials such as paper [30,32,41,42,113,114,115,116,117,136,137,138,139,140,141,142,143,144,145,146,147,148,149,150,151,152,155,156,157]. Although the literature reports different attempts to improve the performance of EGFETs, mainly acting on high aspect ratio, reduced leakage current, and parasitic capacitance, many devices are based on commercial FET, as reported in Table 6, which highlights how the development of EGFET sensors over the years has preferred the use of off-the-shelf components because of the easier fabrication process and lower cost. Though off-the-shelf components ensure easier and faster sensor development, a custom electronic interface can improve the overall performance of the EGFET biosensor (e.g., noise, sensitivity, power consumption) for different specific applications.

As far as pH sensors are concerned, sensitivity does not show significant changes in the reviewed literature, ranging from 45 mV/pH for ZnO nanorod layer [106] to 62.87 mV/pH for the most expensive PdO sensing element [113]. The problem of threshold voltage drift seems to represent a bottleneck ranging from 0.04 mV/h [36] to 13.2 mV/h [92]. On the other hand, the sensitivity of urea and glucose sensors based on EGFET is mostly affected by the sensitive layer leading to a high range of variability. There are many interesting applications and opportunities for the successful development of EGFET-based bio/chemosensors, such as drug discovery, clinical diagnosis of diseases, biomedicine, food safety and processing, environmental monitoring, etc. [51,89,90,177,178]. In particular, the development of disposable and portable detection systems is often preferred over sensitive laboratory-based techniques. Interesting examples are continuous glucose monitoring in diabetic patients, remote sensing of airborne bacteria in counter-bioterrorist activities, and routine monitoring of analytical parameters. In addition, the detection of specific DNA sequences and the study of gene polymorphisms are fundamental to the rapid detection of genetic mutations and sequencing of the genome, offering the possibility of performing a reliable diagnosis even before any disease symptoms appear. Apart from this positive note, very stringent laboratory standards, guidelines, and regulations require that EGFET development should strongly fit the specific demand. Increasing efforts in FET design could be a key point in making a significant contribution to the development of EGFET-based chemical sensors, which could be one reason for industry to invest in more reliable chemical sensors.

## Figures and Tables

**Table 1:** Electronic materials used for working electrodes.

Refs.	Substrate	Functionalization	Target	Method of Immobilization
[59]	Au/Ag–NWs	HRP	Hydrogen peroxidase	Covalent bonding
[60]	Carbon/ZnO	Hb		Covalent bonding
[61]	GC/Ag NPs/MWNTs	Hb		Entrapment
[62]	Graphene/Au–NPs	Enzyme-free		
[63]	Nafion modified GC/CNT	Enzyme-free		
[64]	Au/Ag–NCs	HRP/GOx	Glucose	Entrapment
[65]	ITO/CS–PPy Au–NPs	GOx		Entrapment
[66]	Ag/CNT/CS	GOx/HRP		Layer technique
[67]	BDD/graphene/Pt–NPs	GOx		Adsorption
[68,69,70,71,72]	Si/VACNFs	GOx/HRP		Adsorption
[73]	Graphite NPs	GOx		Covalent bonding
[74]	Pt/Pt–NPs–PPy	SOx	Sulfite	Entrapment
[75]	ITO/PEDOT:PSS	TPM	Dopamine, ascorbic acid	CVD
[76]	FTO/GONPs–PPy	BOx	Bilirubin	Entrapment
[77]	GC	DHB	Adenine dinucleotide	Potential activation
[78]	BDD/MWCNTs	Tyrosinase	Bisphenol A	Entrapment
[79]	GC/PEDOT/MWCNTs	SOD	Wine antioxidants	nr

NW, nanowire; GC, glassy carbon; NP, nanoparticle; MWNT, multiwalled carbon nanotube; CNT, carbon nanotube; NC, nanocube; CS, chitosan; PPy, polypyrrole; BDD, boron-doped diamond; VACNF, vertically aligned carbon nanofiber; PEDOT:PSS, poly(3,4–ethylenedioxythiophene) polystyrene sulfonate; GONP, graphene oxide nanoparticle; HRP, horseradish peroxidase; Hb, hemoglobin; GOx, glucose oxidase; BOx, bilirubin oxidase; SOx, sulfite oxidase; TPM, 3–(trichlorosilyl) propyl methacrylate; CVD, chemical vapor deposition; DHB, 3,4–dihydroxybenzaldehyd; SOD, superoxide dismutase; nr, not reported.

**Table 2:** Main characteristics of some types of pH sensors.

Ref.	Sensitive Material	Sensitivity (mV/pH)	Range	Linearity (%)	Drift (mV/h)	Hysteresis (mV)	Reference Electrode	Sensitive Area	FET Device	Type
[30]	ITO	58	2–12	nr	nr	9.8	SCE	6 mm^2^	CD4007UB	P
[32]	SnO_2_	56–58	2–12	nr	nr	nr	SCE	nr	CD4007UB or LF356N	P
[41]	TiO_2_	59.89	1.8–12	93.50	0.041692–2.6007	5.3–9	Ag/AgCl	1 cm^2^	NDP6060L	P
[42]	V_2_O_5_	58.1 ± 0.8	2–10	nr	nr	nr	nr	nr	CD4007UB	P
[92]	ITO/PET	50.1 ± 1.7	2–12	98.5	13.2	nr	Ag/AgCl	Π × 2^2^ mm^2^	CD4007CN	P
[94]	ITO/PET	45.9–52.3	2.1–12.1	98.3–99.6	nr	nr	Ag/AgCl	Π × 2^2^ mm^2^	CD4007UB	P
[95]	SnO_2_	59.3	2–9.4	nr	nr	nr	Ag/AgCl	nr	LT1167–I.A.	P
[101]	AZO	57.95	1–13	99.98	1.27	4.83	Ag/AgCl	2 × 2 mm^2^	CD4007UB	A
[104]	ITO/SiO_2_/Nb_2_O_5_	59.2	3–13	99.48	2%	1.83%	Ag/AgCl	20 × 20 mm^2^	IC4007	P
[106]	ZnO nano-array	45	4–12	nr	nr	nr	Ag/AgCl	Π × 2.5^2^ mm^2^	CD4007UB	P
[108]	SnO_2_/SiO_2_/glass	58	1–9	nr	nr	nr	Ag/AgCl	1.5 × 1.5 mm^2^	LT1167–I.A.	P
[109]	SnO_2_/ITO/PET	53.8–58.7	2–12	nr	nr	nr	Ag/AgCl	nr	LT1167–I.A.	P
[113]	PdO	62.87 ± 2	2–12	99.97	2.32	7.9	Ag/AgCl	0.25 cm^2^	CD4007UBE	P
[114]	InGaZnO	59.5	2–10	99.7	3–9	nr	Ag/AgCl	nr	CD4007	P
[115]	Glass	55	2–12	nr	nr	nr	nr	nr	CD4007UB	P
[116]	CNT	50.9	3–13	99.78	nr	nr	nr	1 × 2 cm^2^	nr	P
[117]	FTO	54.10	2–12	nr	nr	nr	nr	nr	CD4007UB	P

PET, polyethylene terephthalate; AZO, aluminum-doped ZnO; P, potentiometric; A, amperometric; I.A., instrumentation amplifier; nr, not reported.

**Table 3:** Main characteristics of representative urea sensors.

Ref.	Sensitive Material	Sensitivity	Range (mM)	Linearity (%)	Reference Electrode	Sensitive Area	FET Device	Type
[94]	ITO/PET	21.2 mV/pCurea	1.5–10	96.5	Ag/AgCl	Π × 2^2^ mm^2^	CD4007UB	P
		49.7 mV/pCurea		99.0				
		62.4 mV/pCurea		98.6				
[95]	SnO_2_/ITO	nr	0.05–20		Ag/AgCl	nr	LT1167–I.A.	P
[109]	SnO_2_/ITO/PET	nr	0.04–0.33	97	Ag/AgCl	nr	LT1167–I.A.	
[117]	FTO	8.92 μA/pCurea	0.01–300	nr	nr	nr	CD4007UB	A

P, potentiometric; A, amperometric; I.A., instrumentation amplifier; nr, not reported.

**Table 4:** Main characteristics of representative EGFET-based glucose sensors.

Ref.	Electrode	Sensitivity	Range (mM)	Linearity (%)	Drift (mV/h)	Hysteresis (mV)	Reference Electrode	Sensitive Area (μm^2^)	FET Device	Type
[101]	AZO	60.5 μA·mM^−1^·cm^−2^	0–13.9	99.96	1.27	4.83	Ag/AgCl	2 × 2·10^6^	CD4007UB	A
[135]	Au	−61.6 mV/decade	0.125–1	99.60	nr	nr	Ag/AgCl	10 × 10	0.6 μm CMOS	P
[136]	PPI/NiTsPc	nr	0.05–1	nr	nr	nr	Ag/AgCl	nr	AD620 I.A.	P
[137]	Au	−58 mV/decade	0.1–2	99.97	0.50	nr	Ag/AgCl	20 × 56	32 × 32 array 1.2 μm CMOS	P
[138]	Ru-doped TiO_2_	320 μV/(mg/dL)	5.55–27.55	99.50	nr	nr	Ag/AgCl	2 × 2·10^6^	LT1167 I.A.	P
[139]	ZnO	20.33 μA·mM^−1^·cm^−2^	0.5–10	nr	nr	nr	Ag/AgCl	nr	CD4007UB	P
[140]	ZnO nanorods	nr	0.01–5	nr	nr	nr	Ag/AgCl	nr	Glass FET	P

P, potentiometric; A, amperometric; I.A., instrumentation amplifier; nr, not reported; PPI/NiTsPc, poly(propylene imine) dendrimer/nickel tetrasulphonated phthalocyanine.

**Table 5:** Sensors of calcium ions.

Ref.	Sensitive Material	Sensitivity	Range	Linearity (%)	Reference Electrode	Sensitive Area (mm^2^)	FET Device
[146]	RuO_2_	32.5 mV/pCa	pCa0–pCa2	97.6	Ag/AgCl	nr	NMOS
[147]	Ru-doped TiO_2_ or RuO_2_	29.65 mV/pCa	pCa0–pCa3	99.9	Ag/AgCl	nr	CD4007UB
[148]	PVC	25.02 mV/pCa	0.001–1 mM	99.65	Ag/AgCl	nr	CD4007UB
[149]	ZnO nanorods	26.55 mV/decade	0.001–100 mM	nr	Ag/AgCl	Π × 0.25^2^	nr

PVC, polyvinyl chloride; nr, not reported.

**Table 6:** FET device characteristics for EGFET sensors.

Refs.	FET Device	Model	CMOS Process (µm)	Main Features	W/L (µm/µm)
[158]	NC	N/A	nr	SOI-FET working in parasitic Bipolar Junction Transistor (BJT) operation method	nr
[159]	NC	N/A	0.5	CMOS–DPDM n–well	600/20
[30,32,42,92,94,101,104,105,113,114,117,139,147,148,160,161,162,163,164,165,166,167]	C	CD4007UB	nr	CMOS dual complementary pair plus inverter	nr
[41,168,169]	C	NDP6060L	nr	*n*–Channel logic level enhancement mode FET	nr
[170,171]	C	HEF4007	nr	Dual complementary pair and inverter	nr
[172]	C	BS170	nr	*n*−Channel MOSFET	9700/2
[173]	NC	N/A	0.16	Differential source follower	8/2
[174]	NC	N/A	0.35	Rectangular p-type MOSFET	18/1
[175]	NC	N/A	0.6	Pt and Au gate MOSFET	100/10
[95,108,109,138]	C	LT1167	nr	Instrumentation amplifier	nr
[176]	C	2SK246Y	nr	*n*-Channel junction FET	nr

C, commercial FET device; NC, noncommercial FET device; DPDM, double poly double metal; nr, not reported; N/A, not applicable.
